# Heparin-binding protein is significantly increased in acute pancreatitis

**DOI:** 10.1186/s12876-021-01910-6

**Published:** 2021-08-28

**Authors:** Martina Sjöbeck, Hanna Sternby, Heiko Herwald, Henrik Thorlacius, Sara Regnér

**Affiliations:** 1 Department of Surgery, Clinical Sciences, Malmö, Skåne University Hospital, Lund University, 205 02 Malmö, Sweden; 2 Division of Infection Medicine, Department of Clinical Sciences, Lund University, Lund, Sweden

**Keywords:** Acute pancreatitis, Heparin-binding protein, Biomarkers

## Abstract

**Background:**

Most patients with acute pancreatitis (AP) experience mild, self-limiting disease with little or no need for hospital care. However, 20–25% of patients develop a more severe and potentially life-threatening condition with progressive systemic inflammatory response syndrome (SIRS) and multiorgan failure, resulting in high morbidity and mortality rates. Predicting disease severity at an early stage is important, as immediate supportive care has been demonstrated to reduce the incidence of SIRS and organ failure, improving patient outcome. Several studies have demonstrated elevated levels of heparin-binding protein (HBP) in patients with sepsis and septic shock, and HBP is believed to play a part in endothelial dysfunction leading to vascular leakage. As HBP levels increase prior to other known biomarkers, HBP has emerged as a promising early predictor of severe sepsis with organ dysfunction.

**Methods:**

Patients admitted to Skåne University Hospital in Malmö between 2010 and 2013 fulfilling the criteria for AP were identified in the emergency department and prospectively enrolled in this study. The primary outcome was measured levels of HBP upon hospital admission in patients with confirmed AP. Correlations among HBP concentrations, disease severity and fluid balance were considered secondary endpoints. The correlation between HBP levels and fluid balance were analysed using Pearson correlation, and the ability of HBP to predict moderately severe/severe AP was assessed using a receiver operating characteristic (ROC) curve.

**Results:**

The overall median HBP level in this study was 529 (307–898) ng/ml. There were no significant group differences in HBP levels based on AP severity. Fluid balance differed significantly between patients with mild versus moderately severe and severe pancreatitis, but we found no correlation between HBP concentration and fluid balance.

**Conclusions:**

HBP levels are dramatically increased in patients with AP, and these levels far exceed those previously reported in other conditions. In this study, we did not observe any significant correlation between HBP levels and disease severity or the need for intravenous fluid. Additional studies on HBP are needed to further explore the role of HBP in the pathogenesis of AP and its possible clinical implications.

**Supplementary Information:**

The online version contains supplementary material available at 10.1186/s12876-021-01910-6.

## Background

Most patients with acute pancreatitis (AP) present with mild, self-limiting disease, with little or no need for hospital care. However, 20–25% of patients develop a more severe and potentially life-threatening condition with progressive systemic inflammatory response syndrome (SIRS) and multiorgan failure, resulting in high morbidity and mortality rates [1–3].

Therefore, early recognition of patients with potentially severe disease is crucial. Additionally, immediate supportive care has been demonstrated to reduce the incidence of SIRS and organ failure, improving patient outcomes. The role of fluid resuscitation in acute pancreatitis has recently gained increasing interest. Counteracting hypotension and haemoconcentration using early fluid administration is believed to preserve microcirculation in the pancreas, preventing further damage and necrosis [4, 5]. However, there are controversies regarding the optimal rate and volume of fluid administration in these patients. Therefore, a method for analysing the fluid requirement of each individual patient would be of great clinical value [6–8].

The inflammatory response plays a key role in the pathogenesis of acute pancreatitis, particularly in the more severe forms, and numerous mediators have been investigated as potential prognostic biomarkers [9, 10]. Although some have shown promising results in predicting severe pancreatitis upon hospital admission, none of them have proven sufficient to be incorporated into routine clinical practice [11–13].

Heparin-binding protein (HBP), also known as CAP37 and azurocidin, is a glycoprotein stored in azurophilic granules and secretory vesicles in neutrophils that is released upon neutrophil activation early in the systemic inflammatory response [14].

HBP serves as a potent chemoattractant for monocytes, fibroblasts, and T-cells and enhances the inflammatory response by stimulating the production of tumour necrosis factor (TNF)-alpha, interleukin (IL)-1 and IL-6 [15–20].

In addition, HBP has been shown to increase endothelial cell permeability, leading to vascular dysfunction and plasma fluid leakage in sepsis and in other inflammatory disorders [18, 19, 21, 22].

Several studies have reported elevated levels of HBP in patients with sepsis and septic shock [23–25]. Because HBP levels increase prior to other known biomarkers (*e.g.,* C-reactive protein (CRP), procalcitonin, white blood cell count (WBC) and lactate), it has emerged as a promising early predictor of severe sepsis with organ dysfunction [25]. Interestingly, HBP elevation has been found not only in septic shock but also in patients who develop circulatory failure for other reasons [26–28].

In AP, endothelial dysfunction, plasma fluid leakage and the need for substantial volumes of intravenous fluids to maintain adequate circulation resemble the pathophysiology of sepsis and septic shock. In both cases, neutrophil recruitment and activation play a crucial role in the aggravated inflammatory response. Given the role of HBP in endothelial dysfunction in sepsis, we hypothesized that HBP might also play a crucial role in the development of AP.

Therefore, the aim of this study was to investigate whether the concentration of HBP is elevated in AP and whether HBP can be used as an early predictive biomarker for severe disease. Furthermore, we examined whether HBP represents a biomarker for fluid loss during acute pancreatitis.

## Methods

### Patients and study design

As has been previously described, all patients > 18 years of age diagnosed with AP and admitted to the Department of Surgery, Skåne University Hospital, Malmö, Sweden, from January 2010 to September 2013 were prospectively and consecutively enrolled in a research database [11, 29]. Patients who did not understand information provided in Swedish or with a symptom duration exceeding 72 h were excluded. This study was approved by the Ethics Committee for Clinical Research at Lund University, Sweden (2009/415). Informed consent (oral and written) was obtained from all participants in the study.

The diagnosis of AP was determined at fulfilment of two out of the following three criteria: (1) acute characteristic upper abdominal pain, (2) serum amylase ≥ 3 times the upper reference limit and/or (3) characteristic findings on computed tomography, ultrasound or magnetic resonance imaging.

Clinical data were obtained from patients at inclusion and retrospectively from medical charts [11, 29]. Patients were retrospectively classified as having mild, moderately severe or severe pancreatitis according to the Revised Atlanta Classification of 2012 [30]. Fluid balance was retrospectively obtained from separate fluid charts that were used in routine practice in the surgical wards. Included in the fluid balance were intravenous (iv) and oral fluid input, urinary output, vomiting, fluid loss through gastric tube and IV diuretic administration.

The primary end point was measured levels of HBP upon hospital admission in patients with confirmed AP. The correlation between HBP concentration and disease severity, as well as HBP concentration and fluid balance, were regarded as secondary endpoints.

### Blood samples

EDTA plasma samples were obtained upon admission to the hospital, centrifuged at 2000 rpm for 10 min (25 °C) and stored at − 80 °C until analysis. HBP concentration was determined by enzyme-linked immunosorbent assay (ELISA), as previously described by Tapper et al. [14]. In addition, CRP was analysed using standard methods at the Department of Clinical Chemistry, Skåne University Hospital, Malmö, Sweden.

### Statistical analysis

Data are presented as the mean ± standard deviation (SD) and median with interquartile range (IQR) as appropriate. Proportions were compared using the chi-squared test. With the population stratified into three groups, variables were analysed using one-way analysis of variance (ANOVA) or Kruskal–Wallis test when appropriate. Post hoc pairwise comparisons were adjusted for multiple comparisons using the Bonferroni correction. Since this study included a limited number of patients with severe pancreatitis, we chose to analyse the discriminatory ability of HBP by dichotomizing mild and moderately severe/severe disease. The stratifying capacity of HBP was assessed using a receiver operating curve (ROC), and the results are presented as the area under the curve (AUC). The correlation between HBP levels and fluid balance was analysed using the Pearson correlation. A multivariable logistic regression was performed to assess the independent effect of HBP concentration (in relation to fluid balance, diuretics administered and symptom duration) on disease severity, and the results are presented as odds ratios (ORs) and 95% confidence intervals (CIs). A *p* value < 0.05 was considered statistically significant. All statistical analyses were performed using IBM SPSS Statistics (IBM SPSS Statistics for Mac (2017), version 26. Armonk, NY: IBM Corp).

## Results

### Patient characteristics

A total of 260 patients with AP were included in our research database, and plasma material for HBP analysis was available for 204 of them. In two cases, the analysis failed due to an inadequate amount of plasma, leaving a study cohort of 202 patients.

The baseline characteristics of the study group are presented in Table 1. The mean age was 63.8 ± 18.7 years, and 50% of patients were female. In 59% of cases, patients presented with biliary pancreatitis, 13% were alcohol induced, and 10% were idiopathic, whereas the remaining cases were caused by other mechanisms (*e.g.,* post-endoscopic retrograde cholangiopancreatography (ERCP), tumours, strictures, hypercalcaemia and hyperlipidaemia). In total, 4% of cases were classified as severe AP, 19% as moderately severe and 77% as mild, according to the Revised Atlanta classification [30]. One patient in the mild group was admitted to the intensive care unit (ICU) due to delirium tremens.

**Table 1:** Baseline characteristics

	All (*n* = 202)	Mild (*n* = 155)	Moderately severe (*n* = 38)	Severe (*n* = 9)	p value	Missing
Gender n (%)					0.751	0
Male	102 (50)	76 (49)	21 (55)	5 (56)		
Female	100 (50)	79 (51)	17 (45)	4 (44)		
Age	63.8 ± 18.7	63.0 ± 19.0	65.6 ± 18.5	75 ± 12.0	0.155	0
BMI (kg/m^2^)	25.5 (23.2–29.8)	25.2 (22.9–29.7)	26.6 (24.3–31.0)	24.5 (22.6–30.1)	0.207	0
Aetiology n (%)					0.161	0
Biliary	119 (59)	95 (61)	21 (55)	3 (33)		
Alcohol	27 (13)	17 (11)	9 (24)	1 (11)		
Idiopathic	21 (10)	14 (9)	4 (11)	3 (33)		
Other	35 (19)	29 (19)	4 (11)	2 (22)		
Onset to admission *(h)	9 (3–24)	9 (4–25)	8 (3–24)	8 (0–19)	0.634	2

Values are expressed as the median and interquartile range (IQR), except for age, which is presented as the mean ± standard deviation (SD). Classification of groups according to revised Atlanta Classification

*Onset of symptom until admission to hospital

Age, body mass index (BMI) and symptom duration were similar across the three groups, whereas CRP on the day of admission was 28 (11–59) mg/L, 30 (19–97) mg/L and 122 (83–170) mg/L in the mild, moderately severe and severe groups, respectively (*p* = 0.004).

In total, 5% of the patients were admitted to the ICU—1% in the mild group, 6% with moderately severe pancreatitis and 75% of patients with severe pancreatitis (*p* < 0.001) (Table 2). There were no deaths among patients with mild or moderately severe pancreatitis, whereas 56% of patients with severe pancreatitis succumbed to the disease.

**Table 2:** Patient characteristics according to AP severity

	All (*n* = 202)	Mild (*n* = 155)	Moderately severe (*n* = 38)	Severe (*n* = 9)	p-value
Organ failure (n, %)	22 (11)	0 (0)	13 (34)	9 (100)	< 0.001
ICU (n, %)	9 (5)	1 (1)	2 (6)	6 (75)	< 0.001
Mortality (n, %)	5 (3)	0 (0)	0 (0)	5 (56)	< 0.001
HBP (ng/ml)	529 (307–898)	527 (301–887)	529 (338–955)	640 (383–1465)	0.474
CRP day of admission (mg/L)	29 (12–65)	28 (11–59)	30 (19–97)	122 (83–170)	0.004

Values are expressed as medians and interquartile ranges (IQRs). Classification of groups according to revised Atlanta classification

Fluids administered on the day of hospital admission are presented in Table 3. The majority of patients received lactated ringers and glucose as initial fluid resuscitation.

**Table 3:** Fluids given on the day of admission

	Mild	Moderately severe/severe	*p* value
Glucose 5% (ml)	1441 ± 604 (100)	1558 ± 555 (31)	0.338
Rehydrex®* (ml)	1108 ± 421 (26)	1500 ± 756 (8)	0.197
Normal saline (ml)	1063 ± 487 (12)	1850 ± 0 (1)	0.148
Lactated ringer (ml)	1741 ± 754 (118)	2238 ± 908 (38)	< 0.001
Colloid (ml)	750 ± 112 (6)	500 ± 0 (4)	0.111

Values are given as the mean ± standard deviation and are expressed in millilitres (ml) and n (number of patients)

*Rehydrex® (Fresenius Kabi, Sweden)

### HBP measurement

**Fig. 1:**
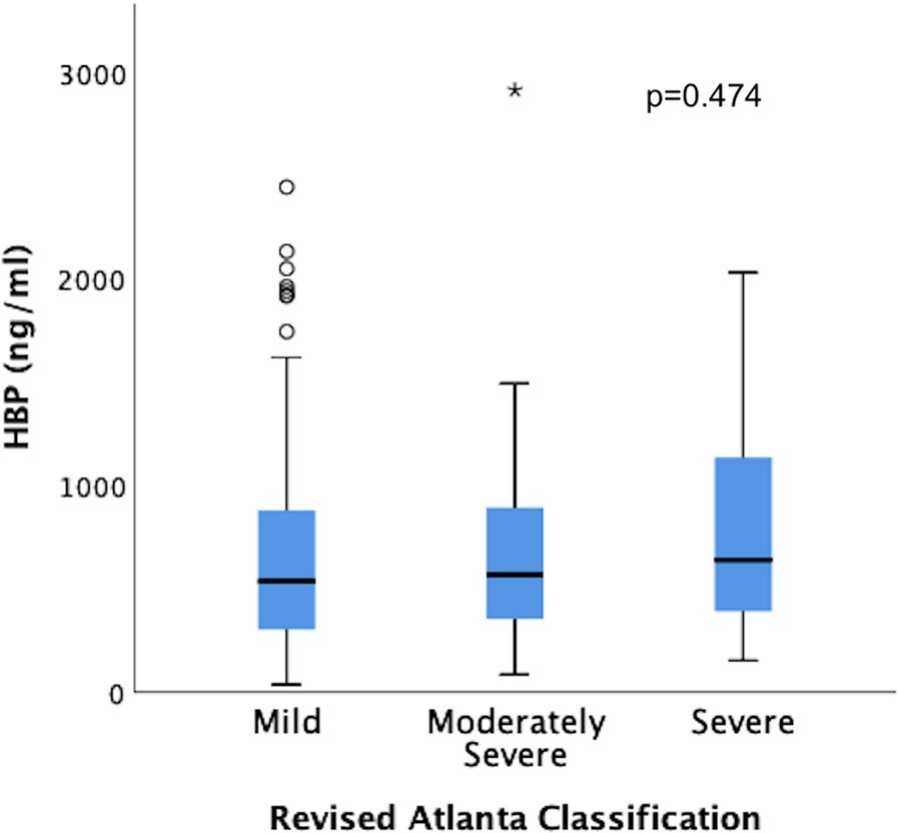
Box plot illustrating the median HBP concentration in the different groups according to the Revised Atlanta Classification

**Fig. 2:**
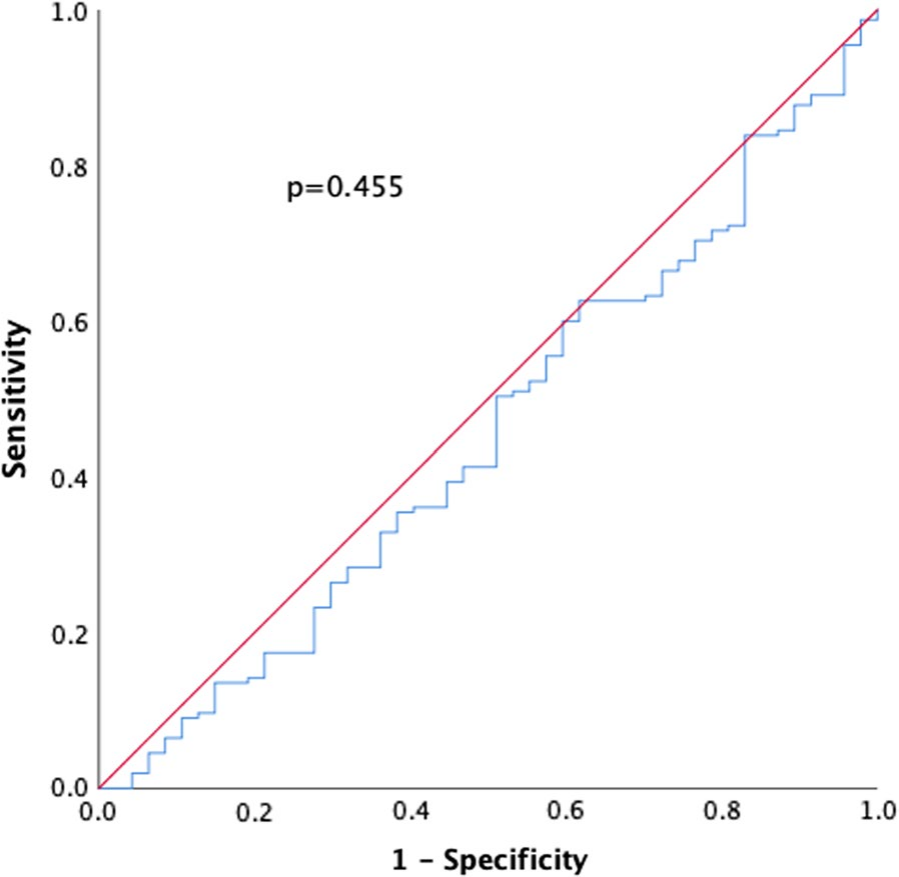
Receiver operating characteristic (ROC) curve illustrating the ability of HBP to discriminate between mild and moderately severe/severe acute pancreatitis

The overall median HBP concentration in this study was 529 (307–898) ng/ml. In mild pancreatitis, the median HBP level was 527 (301–887) ng/ml; in moderately severe cases, it was 529 (338–955) ng/ml; and in the severe group, the median HBP was 640 (383–1465) ng/ml (*p* = 0.474) (Fig. 1). ROC analysis testing the performance of HBP to discriminate between mild and moderately severe/severe AP resulted in an AUC = 0.455 (Fig. 2). When analysing HBP in patients admitted to the hospital < 12 h after symptom onset, we found an HBP concentration of 565 (298–913) ng/ml in mild AP and 529 (368–1102) ng/ml in patients with moderately severe/severe disease (*p* = 0.531). In patients who were admitted > 12 h from the start of symptoms, the median HBP concentration was 508 (302–827) ng/ml in mild AP and 413 (333–1031) ng/ml in moderately severe/severe AP (*p* = 0.493).

We identified significant differences in fluid balance between patients with mild disease compared to those with moderately severe and severe AP (Table 4). However, there was no correlation between HBP concentration and fluid balance on the day of admission (correlation coefficient 0.031, *p* = 0.712) or day one (− 0.010, *p* = 0.899) or two (0.056, *p* = 0.475) after admission. When only those patients who did not receive diuretics were analysed, there was still no correlation between HBP concentration and fluid balance. Additionally, there was no correlation between HBP concentration and the amount of fluid given/hour on the day of admission (correlation coefficient − 0.022, *p* = 0.776).

**Table 4:** Fluid balances day 0–2 according to AP severity grade

	Mild (*n* = 155)	Moderately severe (*n* = 38)	Severe (*n* = 9)	*p* value (mild vs. moderate)	*p* value (mild vs. severe)	*p* value (moderate vs. severe)	Missing
Fluid balance Day 0	1623 (900–2301)	2341 (1458–3092)	2707 (1950–2828)	0.002	0.106	1.000	58
Fluid balance Day 1	453 (− 537–1364)	1750 (548–2438)	3740 (750–6640)	0.001	0.001	0.450	29
Fluid balance Day 2	− 83 (− 600–600)	510 (− 538–1265)	2260 (60–3830)	0.200	0.007	0.157	39

Values are expressed as medians and interquartile ranges (IQRs). All p values were corrected for multiple comparisons using the Bonferroni method. Fluid is expressed in millilitre (ml)

Furthermore, multivariable logistic regression did not identify HPB as independently associated with moderately severe/severe AP (OR; 1.000, 95% CI 0.999–1.001, *p* = 0.742) (Additional file 1: Table S1). However, both fluid balance on the day of admission (OR; 1.010, 95% CI 1.004–1.016, *p* = 0.002) and the need for diuretics (OR; 3.600, 95% CI 1.502–8.632, *p* = 0.004) were associated with moderately severe/severe AP independent of HBP concentration or symptom duration.

## Discussion

To the best of our knowledge, this is the first study to analyse HBP levels in AP. Herein, we demonstrate that the concentration of HBP was dramatically elevated in all patients with AP and that HBP levels far exceeded those previously reported in other conditions. However, although variation in fluid balances corresponded to different severity levels of pancreatitis, this did not correlate with significant differences in HBP levels at admission.

Initial assessment of patients with AP remains a clinical challenge. A number of promising biomarkers and scoring systems have been shown to aid in the prediction of disease severity, but none of them have yet been translated into general clinical use [11–13, 31]. Therefore, management of these patients still relies to a large extent on blunt methods, including monitoring of vital signs and fluid balance, using fluid charts and urinary output.

Several studies have shown the importance of early fluid resuscitation for reducing systemic inflammation and organ failure, which improves patient outcome [4, 5, 32, 33]. The majority of patients experience mild disease [1, 3], many of whom are overtreated and thus subjected to unnecessary risks of fluid overload, hospital-related infections and discomfort, along with the economic burden to the healthcare system[34, 35].

HBP has shown promising results as an early biomarker for predicting hypotension and organ failure in patients with sepsis [25]. Part of this is believed to be due to HBP’s involvement in vascular dysfunction, with HBP’s interaction with the endothelial surface contributing to vascular leakage and oedema formation [18, 19, 21, 22]. In acute pancreatitis, a similar inflammatory response and endothelial dysfunction are observed, with both a local and systemic inflammatory response. In more severe cases, this reaction leads to hypotension and organ dysfunction. As such, HBP could be useful as a tool for estimating the severity of AP, as well as the volume of fluid required in individual patients.

Interestingly, the levels of HBP upon admission were substantially elevated in all patients with AP included in this study. The median value of HBP was 529 (307–898) ng/ml, which far exceeds any previously reported HBP levels. Linder et al. published several studies on HBP in the setting of septic shock, with median HBP levels of 40–65 ng/mL [23, 25]. In cardiac arrest and in acute respiratory distress syndrome (ARDS), similar HBP levels were reported [27, 28].

The extremely high concentrations of HBP, regardless of disease severity, may indicate that HBP plays a key role in the pathophysiology of acute pancreatitis by increasing vascular permeability and promoting fluid loss. HBP is released early in response to activation of neutrophils and binds to proteoglycans on the endothelial surface. Thereafter, by mechanisms that are still not fully established, HBP rearranges the cytoskeleton of endothelial cells, leading to alterations in their permeability. In the setting of acute pancreatitis, trypsin activation triggers recruitment and activation of neutrophils, and in turn, neutrophils cause trypsin activation locally in the pancreatic tissue, resulting in a vicious cycle. This interplay might explain the dramatically increased HBP levels that set AP apart from other diagnoses [36].

Despite demonstrating generally increased levels in AP, this study did not observe any significant difference in HBP levels between the different severity groups, as confirmed by a ROC analysis with an area under the curve (AUC) of 0.455. One should keep in mind that the number of patients presenting with severe pancreatitis in our study was low, comprising only 9 patients. However, this group seems to be representative of severe AP based on exhibiting significantly higher CRP levels than those with mild disease (*p* = 0.004) and a mortality rate of 56% compared to 0% among the remaining patients.

In this study, patients had HBP concentrations of 527 (301–887) ng/ml, 529 (338–955) ng/ml and 640 (383–1465) ng/ml when presenting with mild, moderately severe and severe disease, respectively (*p* = 0.474). Despite the low number of patients with severe AP, the results indicate a wide range of HBP levels, similar to other biomarkers studied in AP [11, 13]. These large variations make HBP less useful as a predictive marker for severity.

On the other hand, our data revealed that fluid balance in the first days differs between AP severity groups, with patients with moderately severe and severe disease requiring more fluid than patients with mild disease. Our findings are consistent with other studies showing that patients with the moderately severe and severe form of AP are in need of more IV fluid [4, 8]. However, to our knowledge, this is the first study to demonstrate that this variable corresponds to a difference in overall fluid balance. A large positive fluid balance, despite diuretics, could give clinicians an early hint that the patient is heading towards a more severe form of the disease. In addition, our multivariable logistic regression showed that fluid input/hour on the day of admission and the use of diuretics were associated with moderately severe/severe AP independent of symptom duration or HBP concentration. However, it is likely that there is a reverse causality between moderately severe/severe AP and these factors, as severe AP leads to loss of excessive fluid volume and multi-organ failure with reduced renal output, triggering the use of diuretics.

Therefore, although appealing in theory, we did not observe any correlation between HBP and fluid balance in our study. The fact that HBP levels did not correlate with fluid balance might indicate that there are additional mechanisms responsible for increasing vascular permeability in pancreatitis.

This study has several limitations, the most important being the small number of patients with severe AP, evidently increasing the risk of type II errors. Furthermore, the fluid balances were collected retrospectively. Although the registration of fluid charts is a well-established routine in our surgical wards, there is always uncertainty in how well urine output is reported. In addition, the volume of fluid given to patients was decided by the physician in charge and not according to a standardized protocol. Therefore, the causality between the amount of fluid given and the level of the disease could be questioned. However, the study is strengthened by its prospective and consecutive inclusion of patients and a well-established protocol for handling blood samples. Furthermore, our HBP analyses were conducted in the same laboratory using the same methods and ELISA chemicals as several key studies in HBP research, reducing the risk of incomparable results [19, 23–25, 27].

More studies are needed to further illustrate the role of HBPs in the pathogenesis of acute pancreatitis and to determine whether HBP can be used as a biomarker for the early detection of patients who will develop more severe disease.

## Conclusions

To our knowledge, this is the first study reporting a distinct general elevation in concentrations of HBP in the setting of AP. Further research should aim to investigate the role of HBP in the pathophysiology of acute pancreatitis and whether HBP can be used as a biomarker for the early detection of specific complications in patients with moderately severe and severe disease.

## Supplementary Information

**Additional file 1. Table S1**: Multivariable logistic regression analysis for identifying independent predictors of moderately severe/severe pancreatitis.

## Data Availability

The datasets used and/or analysed during the current study are available from the corresponding author upon reasonable request.

## Abbreviations

ANOVA: Analysis of variance
AP: Acute pancreatitis
ARDS: Acute respiratory distress syndrome
AUC: Area under the curve
BMI: Body-mass index
CRP: C-reactive protein
ELISA: Enzyme-linked immunosorbent assay
ERCP: Endoscopic retrograde cholangiopancreatography
HBP: Heparin-binding protein
ICU: Intensive care unit
IL: Interleukin
IQR: Inter-quartile range
ROC: Receiver operating curve
SD: Standard deviation
SIRS: Systemic inflammatory response syndrome
TNF: Tumour necrosis factor
WBC: White blood cell count
