# Supplementary material for: Heparin-binding protein is significantly increased in acute pancreatitis
Source: BMC Gastroenterol. 2021 Aug 28;21:337. doi: 10.1186/s12876-021-01910-6 (PMC8403433; doi:10.1186/s12876-021-01910-6)
Supplement: Supplementary file 1 — Additional file 1. Table S1: Multivariable logistic regression analysis for identifying independent predictors of moderately severe/severe pancreatitis. [file 12876_2021_1910_MOESM1_ESM.docx]

**Supplementary Table 1.** Multivariable logistic regression on moderately severe/severe pancreatitis

|  | OR (95% CI) | *p-value* |
| --- | --- | --- |
| HBP | 1.000 (0.999-1.001) | 0.742 |
| Symptom duration | 1.001 (0.977-1.026) | 0.912 |
| Furosemide administered | 3.600 (1.502-8.632) | 0.004 |
| Fluid balance day 0 (ml/h) | 1.010 (1.004-1.016) | 0.002 |
